# The role of electrostatic energy in prediction of obligate protein-protein interactions

**DOI:** 10.1186/1477-5956-11-S1-S11

**Published:** 2013-11-07

**Authors:** Mina Maleki, Gokul Vasudev, Luis Rueda

**Affiliations:** 1 School of Computer Science, University of Windsor, 401 Sunset Avenue, Windsor, Ontario, N9B 3P4, Canada

## Abstract

**Background:**

Prediction and analysis of protein-protein interactions (PPI) and specifically types of PPIs is an important problem in life science research because of the fundamental roles of PPIs in many biological processes in living cells. In addition, electrostatic interactions are important in understanding inter-molecular interactions, since they are long-range, and because of their influence in charged molecules. This is the main motivation for using electrostatic energy for prediction of PPI types.

**Results:**

We propose a prediction model to analyze protein interaction types, namely obligate and non-obligate, using electrostatic energy values as properties. The prediction approach uses electrostatic energy values for pairs of atoms and amino acids present in interfaces where the interaction occurs. The main features of the complexes are found and then the prediction is performed via several state-of-the-art classification techniques, including linear dimensionality reduction (LDR), support vector machine (SVM), naive Bayes (NB) and *k*-nearest neighbor (*k*-NN). For an in-depth analysis of classification results, some other experiments were performed by varying the distance cutoffs between atom pairs of interacting chains, ranging from 5Å to 13Å. Moreover, several feature selection algorithms including gain ratio (GR), information gain (IG), chi-square (Chi2) and minimum redundancy maximum relevance (mRMR) are applied on the available datasets to obtain more discriminative pairs of atom types and amino acid types as features for prediction.

**Conclusions:**

Our results on two well-known datasets of obligate and non-obligate complexes confirm that electrostatic energy is an important property to predict obligate and non-obligate protein interaction types on the basis of all the experimental results, achieving accuracies of over 98%. Furthermore, a comparison performed by changing the distance cutoff demonstrates that the best values for prediction of PPI types using electrostatic energy range from 9Å to 12Å, which show that electrostatic interactions are long-range and cover a broader area in the interface. In addition, the results on using feature selection before prediction confirm that (a) a few pairs of atoms and amino acids are appropriate for prediction, and (b) prediction performance can be improved by eliminating irrelevant and noisy features and selecting the most discriminative ones.

## Background

Gene expression, cell growth, proliferation, signal transduction, cellular motion and gene regulation are some of the essential biological processes in living cells which are controlled by proteins [1]. As a consequence of this, more attention has been drawn to this field of study, in particular, for identification and analysis of interacting proteins and their relevant properties [2,3]. Proteins bind to each other, creating protein-protein interactions (PPIs) through a combination of hydrophobic bonding, van der Waals forces and salt bridges. The strength of these interactions may depend on the size of the binding interface which can be large surfaces, small binding clefts or even a few peptides.

Prediction of PPI types is one of the main challenges when studying protein interactions. There are different types of PPIs and their associated prediction problems, including homo vs. hetero-oligomers based on the similarities between sub-units [4], dimers vs. trimers based on the number of interacting sub-units, transient vs. permanent based on the duration of the interaction [5] and obligate vs. non-obligate based on the stability of the complex [6-9]. Despite obligate and permanent interactions, which are more stable and last for a longer period of time, studying non-obligate and transient interactions is a very difficult problem, because of their instability and short life [10]. We focus on distinguishing between obligate and non-obligate complexes.

Using relevant features or observed properties of protein complexes is essential in performing accurate predictions. As a consequence of this, previous studies in PPI have considered a wide range of relevant properties that can be used for PPI prediction including geometric properties [11], recognition of sites [12], conservation of residues present in the surface of PPIs [13,14], hydrogen bonds and salt bridges on the surface of the proteins [13], solvent accessibility [6,15], hydrophobicity [8,16], sequence-based features [17], desolvation energy [18-20] and recently, electrostatic energy [21]. Electrostatic interactions are one of three types of non-covalent interactions, which occur between electrically charged atoms having both positive and negative interactions [22]. Non-covalent interactions are very common between macromolecules such as proteins. Van der Waal interactions, which occur between any pair of charged atoms that are close to each other, and non-polar interactions, which occur between atoms that do not have any charge, are other two types of non-covalent interactions.

In previous studies, it has been claimed that only a few highly conserved residues are important for protein interactions [23-25]. Moreover, removing irrelevant and redundant features not only can decrease the computational burden, but also may increase the prediction performance [26]. These are the main tasks carried out by specialized machine learning algorithms for feature selection and classification. In this regard, automatic feature selection algorithms have been used in many biological problems such as prediction of tyrosine sulfation and lysine ubiquitination [27,28], prediction of protein-protein interactions [25,29], protein-nucleic acid interactions [30], gene selection [31,32] and gene expression [33]. In this study, a few feature selection methods, including gain ratio (GR), information gain (IG), chi-square (Chi2) and minimum redundancy maximum relevance (mRMR), are applied to score and rank features based on their relevance, and select the top ranked features for prediction of obligate and non-obligate PPIs.

In one of our recent works [21], a model to predict obligate and non-obligate protein interaction types has been presented in which electrostatic energy values for both atom and amino acid pairs present in the interface were considered as the input features of the classifiers. Linear dimensionality reduction (LDR) and a support vector machine (SVM) were applied as the classifiers to predict these types. The prediction results of that study for two well-known datasets, referred to as the ZH [6] and MW [5] datasets, show an impressive accuracy in prediction. For the ZH dataset, an accuracy of 96.18% was achieved by using SVM and electrostatic energy values of amino acid type features, which is much higher than the accuracy obtained by using six interface properties including interface area, interface area ratio, conservation score and gap volume index of NOXClass [6] with 88.52% prediction accuracy (as reported by the authors), 46 solvent accessible and interface area properties of [18] with 81.83% prediction accuracy, 210 features of solvent accessible area of [34] with 92.20% prediction accuracy, and even higher than 210 desolvation energy values for amino acid type features of [18] with 83.21% prediction accuracy. Similarly, applying the proposed scheme on the MW dataset demonstrates that using electrostatic energy values of amino acid type features (95.38% prediction accuracy for SVM) is better than using the four interface features as in [6] (77.96% prediction accuracy), and also better than using 210 desolvation energy properties as in [18] (78.83% prediction accuracy). Generally, the results reported in our previous study [21] implied an increase of at least 5% in prediction performance from previous approaches.

This paper is an extension of the work presented in [21] by incorporating a wider range of classification techniques that include LDR, SVM, naive Bayes (NB) and *k*-nearest neighbor (*k*-NN). Distance cutoff selection approaches are also used for analysis of long-range interactions (ranging from 5Å to 13Å), and feature selection algorithms for identifying relevant physicochemical properties of interacting pairs of atoms and amino acids, including GR, IG, Chi2 and mRMR, and an extended visual analysis. The results confirm that electrostatic energy with distance cutoffs ranging from 9Å to 12Å is the best property to predict obligate and non-obligate PPIs on the basis of the experimental results using different classification methods and different distance cutoffs on two well-known datasets. This is due the fact that using electrostatic energy with a long distance cutoff, atoms on the surface and some atoms buried under the surface may participate in the prediction that lead to excellent classification performance. In fact, the latter is a problem that opens an interesting research avenue in the field. Furthermore, using LDR as the classification scheme, we demonstrate that prediction results are improved by applying feature selection and identifying more relevant and discriminative features, while removing redundant and noisy ones for the two datasets.

## Methods

### Datasets

In this study, we have used the same datasets as those used in [18,25]. The first dataset, referred to as the ZH dataset, was obtained from the study of Zhu *et al. *[6]. It originally contained 62 non-obligate and 75 obligate complexes. Since the electrostatic energy values of some complexes (1cc0 A:E, 1qbk B:C, 1b8a A:B, 1cli A:B, 1qav A:B, 1bkd R:S and 1nse A:B) cannot be computed, they were removed from the ZH dataset. The second dataset, referred to as the MW dataset, was obtained from the study of Mintseris *et al. *[5], and originally contained 209 non-obligate and 115 obligate complexes. Similarly, 24 complexes of the original dataset (1b7y A:B, 1be3 CDEGK:A, 1jb0 AB:C, 1jb0 AB:D, 1jb0 AB:E, 1jro A:BD, 1jv2 A:B, 1k28 A:D, 1kqf A:B, 1ldj A:B, 1m2v A:B, 1mjg AB:M, 1nbw AC:B, 1prc C:HLM, 1bgx HL:T, 1de4 CF:A, 1ezv E:XY, 1is8 ABEJCIDHGF:KLOMN, 1m2o AC:B, 1o94 AB:CD, 1qfu AB:HL, 2hmi AB:CD, 4cpa I:0 and 2q33 A:B) were left out because the electrostatic energy values for all atoms in their interfaces cannot be computed.

### Prediction properties

Different properties can be employed to predict protein interactions and, in particular, types of protein complexes. In our recent study [21], it has been demonstrated that electrostatic energy is a powerful property to predict obligate and non-obligate complexes. Moreover, we have previously shown that desolvation energy is also very effective for prediction of these types of PPIs [18,20]. In this study, electrostatic energy properties are used for prediction of obligate and non-obligate interactions and desolvation energy properties are used for comparison purposes. Our method to obtain these prediction properties are summarized below.

#### Desolvation energy

Considering *e_ij _*as the atomic contact potential (ACP) between the *i^th ^*atom of a ligand and the *j^th ^*atom of a receptor, the total desolvation energy for a protein (Δ*G_des_*) is defined as follows [35]:

(1) $$\text{Δ}G_{des}=\overset{18}{\underset{i=1}{\sum }}\overset{18}{\underset{j=1}{\sum }}e_{ij}*g\left(r_{ij}\right).$$

where all atom pairs (18 different atoms) are considered in the double summation and *g*(*r_ij_*) is a smooth function based on the distance of interacting atoms *i *and *j*. For simplicity, in our comparisons, the value of *g*(*r_ij_*) is 1 for pairs of atoms that are less than the selected distance cutoff apart from each other, and 0 otherwise. Using Eq. (1), the desolvation energy between any pair of ligand and receptor can be calculated. Thus, by following the approach of [36], it is possible to compute the desolvation energy by using different criteria. Desolvation energy values are calculated for atom and amino acid types. More details about the computation of desolvation energy values for atom and amino acid types as features can be found in [20].

#### Electrostatic energy

The main property that we use in this study for predicting obligate and non-obligate complexes is electrostatic energy, because of its role in charged molecules [37]. Electrostatic energy involves a long-range interaction and can occur between charged atoms of two interacting proteins or two different molecules. Moreover, these interactions can occur between charged atoms on the protein surface and charges in the environment. In order to compute electrostatic energy values, PDB2PQR[38] and APBS [39] software packages are used.

For each complex in the datasets, after extracting the structural data from the Protein Data Bank (PDB) [40], PDB2PQR is employed for preparing the structures for electrostatic calculations. Adding missing heavy atoms, placing missing hydrogen atoms and assigning charges are some of the main tasks performed by PDB2PQR. To customize the parameters of PDB2PQR in our experiments, we consider the following parameters: (a) the AMBER forcefield is employed (b) "apbs-input" is specified to create output files with ".in" extension, and (c) "*−−*chain" is also specified to include the chain name in the ".pqr" files. The outputs of this package, a "pqr" file and an "in" file, are the inputs to APBS.

APBS is utilized to compute electrostatic energy values of interactions between solutes in salty and aqueous media. In APBS, the Poisson-Boltzmann equation is solved numerically and electrostatic calculations are performed in a range from ten to million atoms. Before running APBS, the parameters should be set accordingly as detailed in [21].

**Figure 1:**
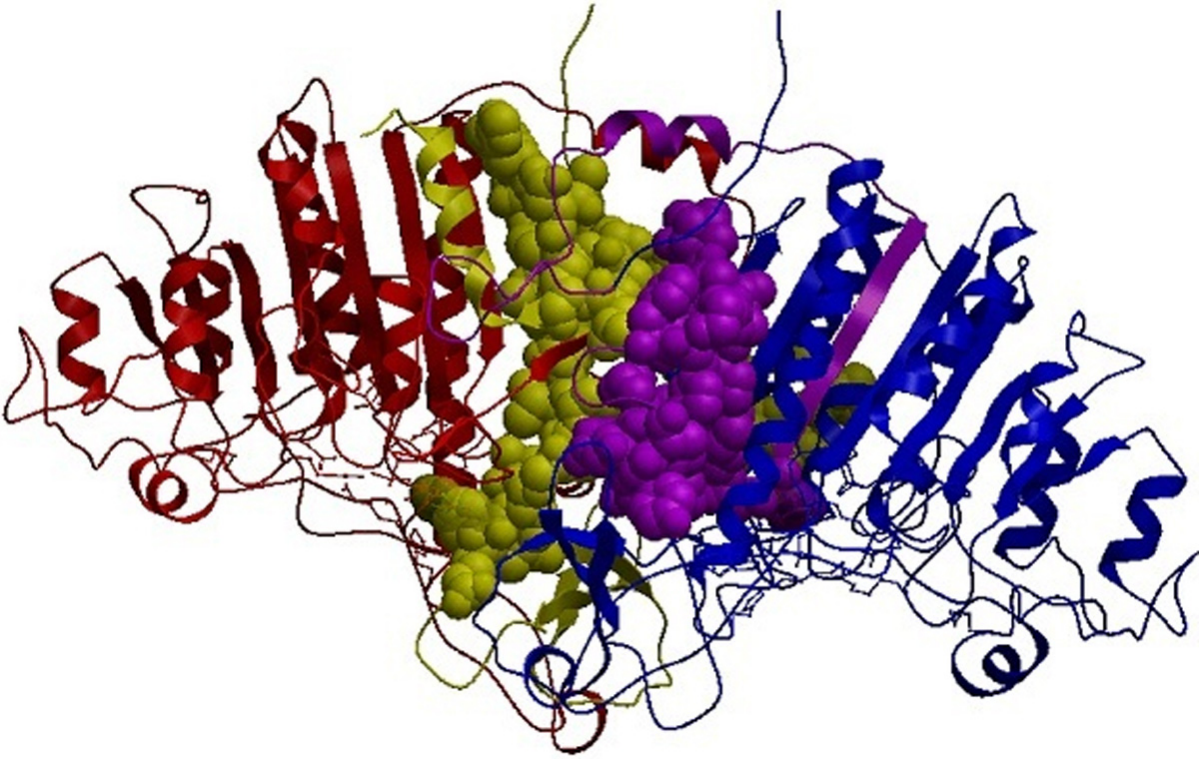
**Quaternary structure of complex 1b8j showing interface atoms for two interacting chains A and B**. Quaternary structure of an obligate complex, PDB-ID 1b8j, visualized with ICM Browser, along with its interacting chains A and B. Positive and negative charges are represented in red and blue respectively. Interface atoms of the interacting chains are represented in yellow and purple, respectively.

To compute the features for classification, first of all, a cutoff distance should be defined. While in most studies, this cutoff, which is the maximum distance between interacting atoms, is considered to be less than 7Å we use cutoffs greater than 7Å. Due to the long-range nature of electrostatic interactions, electrostatic forces towards the stability of the protein complex may be affected by atoms that are under the surface of the proteins. Afterwards, the distances between all atom pairs of interacting chains are computed and those that are lower than our defined cutoff distance are considered as interface atoms. The quaternary structures of chains A (shown in red) and B (shown in blue) of an obligate complex, PDB ID 1b8j, are depicted in Figure 1. The yellow and purple colors indicate atoms that are under the specific cutoff distance and act as interface atoms of chains A and B, respectively. It is clear that a large interface area is taken into account due to the long-range nature of electrostatic interactions.

As in [36], 18 different atom types and 20 different amino acid types were taken into account to calculate the features for prediction. Since the order of the interacting atoms and amino acid pairs is not important, we generated feature vectors for atom type features containing 171 ${(}_{2}^{18}C+18)$ values. Similarly, for amino acid type features, the length of the feature vector 210 ${(}_{2}^{20}C+20)$. Each feature contains the cumulative sum of electrostatic energy values for all pairs of atoms or amino acids of the same type. More details about the computation of electrostatic energy values for atom and amino acid type features are described in [21].

For the ZH and MW datasets, the names of the generated subsets of features for prediction using different feature types (interacting atoms or amino acids) are listed in Table 1.

**Table 1:** Description of datasets used in this study.

Authors	Reference	Atom type	Amino acid type
Mintseris *et al*.	[5]	MW-AT	MW-AA

Zhu *et al*.	[6]	ZH-AT	ZH-AA

By using electrostatic energy for different types of features for the ZH and MW datasets, four subsets of features were generated. In this table, AT stands for atom type features while AA stands for amino acid type features for both datasets of ZH and MW.

### Prediction methods

After finding the features of the complexes of the MW and ZH datasets, a prediction method should be applied to them. In this paper, the prediction is performed via several commonly used classification methods, including LDR, SVM, NB and *k*-NN. More details regarding the applied prediction methods are discussed below.

#### Linear Dimensionality Reduction

The main goal of LDR is to use linear combinations of the original features to generate new features in a lower dimensional space in which classification is, hopefully, more efficient than in the original space. There are different supervised LDR methods, and in this study, the following are considered:

1. Fisher's discriminant analysis (FDA): FDA is a homoscedastic criterion that maximizes the Mahalanobis distance between the means assuming that the covariance matrices are equal.

2. Heteroscedastic discriminant analysis (HDA): HDA is a criterion that starts from the Chernoff distance in original space and takes correlations between random variables to project the data onto a lower dimensional space.

3. Chernoff discriminant analysis (CDA): CDA is a heteroscedastic criterion and aims to maximize the Chernoff distance between random vectors in the transformed space.

LDR is followed by a Bayesian classifier (linear or quadratic). More details about these LDR methods and the corresponding classification tasks can be found in [41].

#### Support Vector Machine

SVMs are well known machine learning techniques used for classification, regression and other tasks. The main goal of the SVM is to find a hyperplane that classifies all the feature vectors into two regions. In most cases, the separating hyperplane is not unique, and hence the SVM chooses the hyperplane that leaves the maximum margin from that hyperplane to the support vectors. Since most classification problems are not linearly separable, using a linear classifier is inefficient. Thus, in order to achieve a more efficient classification, using kernels to map the data onto a higher dimensional space can be useful. There are a number of kernels that can be used in SVM models such as polynomial, radial basis function (RBF) and sigmoid. The effectiveness of the SVM depends on the selection of the kernel, the selection parameters and the soft margin [42]. In addition, sequential minimal optimization (SMO), is a fast learning algorithm that has been widely applied to the training phase of a SVM classifier to solve the underlying optimization problem. In this study, the SMO module of the Waikato Environment for Knowledge Analysis (WEKA) with a polynomial kernel, default parameter settings and 10-fold cross validation is used for performing classification via the SVM [43].

#### k**-Nearest Neighbor**

*k-*NN is one of the simplest classification methods in which the class of each test sample can be easily found by voting on the class labels of its neighbors. To achieve this, after computing and sorting the distances between the test sample and each training sample, the most frequent class label in the first *k *train samples (nearest neighbors) is assigned to the class of the test sample. Determining the appropriate number of neighbors is one of the challenges of this method. In this study, the IBK module of WEKA with Euclidean distance, default parameter settings, and 10-fold cross validation is used for *k*-NN classification [43].

#### Naive Bayes

One of the simplest probabilistic classifiers is NB. Assuming independence of the features, the class of each test samples can be found by applying Bayes' theorem. The basic mechanism of NB is rather simple. The reader is referred to [26] for more details. In this study, the NaiveBayes module of WEKA with default parameters and 10-fold cross validation is used [43].

### Feature selection methods

Feature selection is the process of selecting the best subset of relevant features that represents the whole dataset efficiently and removing redundant and/or irrelevant ones. Applying feature selection before running a classifier is useful in reducing the dimensionality of the data and, thus, reducing the prediction time, while improving the prediction performance by eliminating irrelevant, redundant and noisy features. There are two different ways of doing feature selection: wrapper methods and filter methods [44]. In this study filter-based methods are used in which the quality of the selected features are scored and ranked independently of the classification algorithm and by using some criteria based on their relevance. The following filter-based feature selection methods are used in this study.

#### Minimum Redundancy Maximum Relevance

One of the most widely-used feature selection methods based on mutual information is mRMR [45,46]. In this method, the features are selected and scored based on their relevance and redundancy among other features. A feature with minimum redundancy and maximum relevance and with respect to the class concept is assigned a high score. After assigning a significance score to each feature, a ranking list of all features is generated. In this study, the online mRMR tool [47] with default parameters is used to obtain a complete list of all scored features by mRMR.

#### Information Gain

Information gain (IG) is based on the concept of entropy [44]. The IG value of a feature *X *with respect to class attribute *Y *is calculated as follows:

(2) IG(Y,X)=H(Y)-H(Y|X).

Here, *H*(*Y *) is the entropy of class *Y *and *H(Y|X) *is the conditional entropy of *Y *given *X*, which are calculated by means of the following formulas:

(3) $$H\left(Y\right)=-\underset{y\in Y}{\sum }p\left(y\right){\text{log}}_{2}\left(p\left(y\right)\right),$$

and

(4) $$H\left(Y|X\right)=-\underset{x\in X}{\sum }p\left(x\right)\underset{y\in Y}{\sum }p\left(y|x\right){\text{log}}_{2}\left(p\left(y|x\right)\right),$$

where *p*(*y*) is the marginal probability density function for random variable *Y *and *p(y|x) *is the conditional probability of *Y *given *X*. In this study, the InfoGainAttributeEval module of WEKA is used for feature ranking based on the score of features by measuring the information gain with respect to the class.

#### Gain Ratio

GR attribute evaluation is a well-known feature selection method that is based on the concept of IG and entropy [44]. The GR value of a feature *X *with respect to class attribute *Y *is calculated as follows:

(5) $$GR\left(Y,X\right)=\frac{\left(H\left(Y\right)-H\left(Y|X\right)\right)}{H\left(X\right)}=\frac{IG\left(Y,X\right)}{H\left(X\right)},$$

where *H*(*Y*), the entropy of class *Y *, and *H(Y|X)*, the conditional entropy of *Y *given *X*, are calculated using Eqs. (3) and (4) respectively. A value of GR = 1 indicates that feature *X *is highly relevant and one of the best features to predict class *Y *, while GR = 0 means that feature *X *is not relevant at all. In this study, the GainRatioAttributeEval module of WEKA is used for feature ranking based on the relevance of each feature by measuring its gain ratio with respect to the class.

#### Chi Square

Feature selection via the Chi square test is another, very commonly used method [44]. This method evaluates the relevance of a feature with respect to a class by computing the value of the Chi square statistic. In this study, the ChiSquaredAttributeEval module of WEKA is used to obtain the scored feature vector.

## Results and discussion

To test our proposed method and perform an in-depth analysis of the strength of electrostatic energy as the prediction property, four different classification methods including SMO, *k*-NN, LDR and NB and also four different feature selection methods including IG, GR, Chi2 and mRMR have been used. The performances of the prediction methods are compared in terms of their accuracies, which are computed as follows: *acc *= (*T P *+ *T N *)*/N *, where *T P *and *T N *are the total numbers of true positive (obligate) and true negative (non-obligate) counters over the 10-fold cross-validation procedure, respectively, and *N *is the total number of complexes in the dataset.

### Analysis of prediction properties

In previous works [18-20], it has been shown that desolvation energy is very efficient for prediction of obligate and non-obligate complexes in comparison with solvent accessible and interface area properties. However, in our recent study of [21] and in this work, it has been shown that employing electrostatic energy deliver impressive prediction accuracy.

To validate our previous results and compare the strength of electrostatic and desolvation energies as properties for prediction, SMO, *k*-NN, NB and LDR have been applied for prediction on these two types of features. For the LDR schemes, six different classifiers were implemented and evaluated, namely the combinations of FDA, HDA and CDA with quadratic and linear classifiers; the maximum average classification accuracy for each classifier is reported for each dataset. For SVM, *k*-NN and NB, the classification modules of WEKA have been used with default parameters in a 10-fold cross-validation process. The distance cut-offs between atom pairs of interacting chains are 9Å and 7Å for electrostatic and desolvation energies as properties respectively.

The prediction results of SMO, NB, *k*-NN and LDR for atom and amino acid type properties for the ZH and MW datasets with desolvation and electrostatic energies as properties are shown in Table 2. For ZH-AT, the best accuracy by using electrostatic energy is 96.95% with SMO, while by using desolvation energy, accuracy is much lower, 74.34%, with LDR. Also, for ZH-AA, using electrostatic energy leads to 97.70% accuracy with SMO, being more efficient than using desolvation energy with NB, 75.91%. Similarly, the best accuracies for MW-AT, 96.30%, and MW-AA, 98.68%, are obtained using electrostatic energy in comparison with accuracies of 89.44% and 75.15% for both MW-AT and MW-AA respectively by using desolvation energy.

**Table 2:** Comparison of accuracies for electrostatic and desolvation energies as properties.

Dataset	SMO		NB		*k*-NN		LDR	
	
	DE	EE	DE	EE	DE	EE	DE	EE
ZH-AT	72.52%	**96.95%**	72.52%	94.65%	64.12%	95.42%	74.34%	95.42%

ZH-AA	66.42%	**97.70%**	75.91%	92.37%	54.74%	96.18%	72.13%	93.89%

MW-AT	77.30%	96.04%	77.96%	89.44%	74.43%	95.71%	78.95%	**96.30%**

MW-AA	73.93%	**98.68%**	72.39%	90.10%	57.36%	98.68%	75.15%	92.08%

Prediction accuracies of SMO, NB, *k*-NN and LDR for all datasets of ZH and MW (ZH-AA, ZH-AT, MW-AA and MW-AT) using desolvation energy (DE) and electrostatic energy (EE) as properties.

Generally, from the table, it can be concluded that electrostatic energy yields much more efficient prediction than desolvation energy, on the basis of the experimental results shown here using different classification methods. In addition, for most subsets of features, SMO performs better than *k*-NN, NB and LDR, for both desolvation and electrostatic energies.

**Figure 2:**
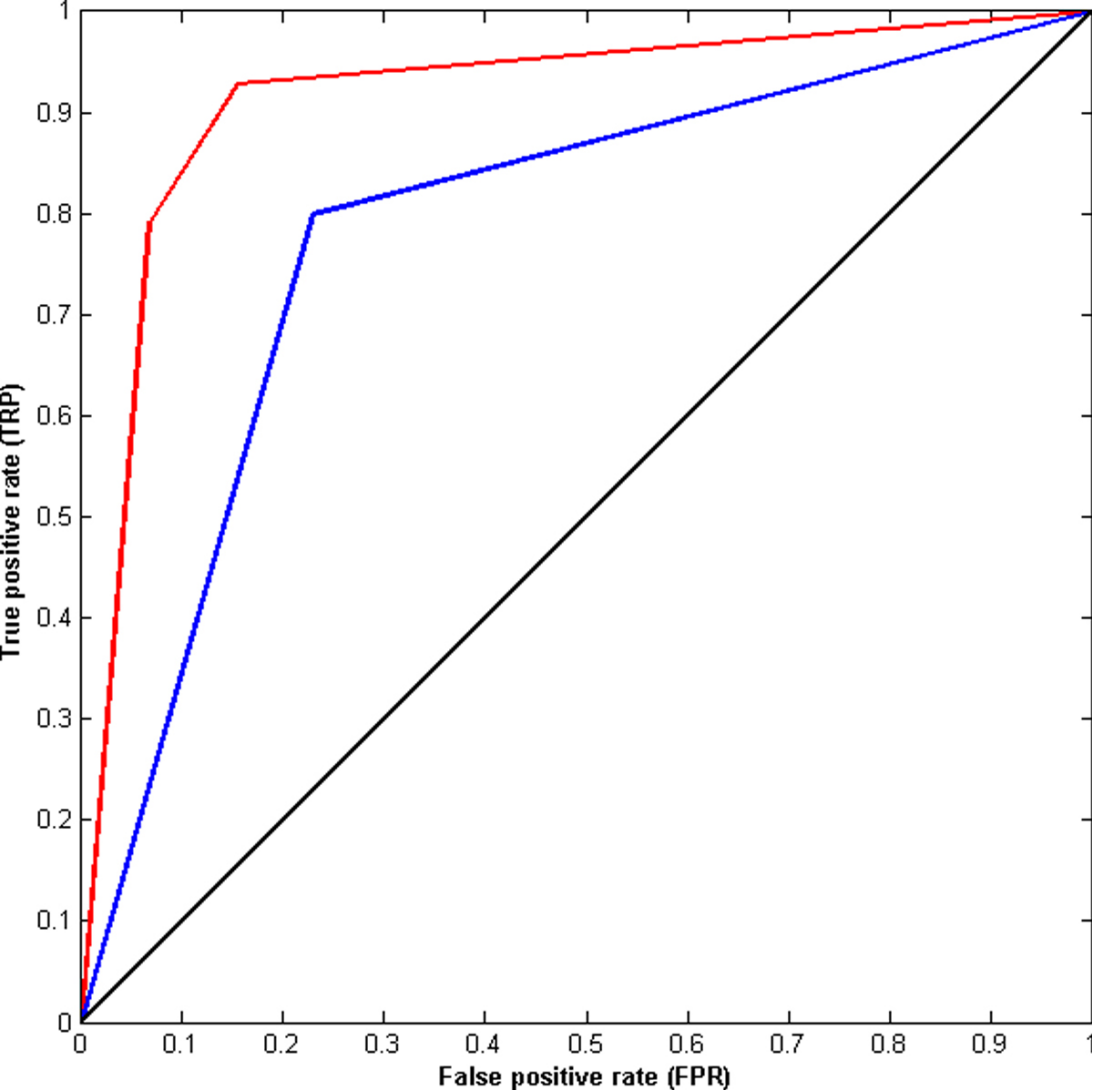
**ROC curve for the MW-AT dataset**. ROC curves for the MW-AT dataset using desolvation energy (blue line) and electrostatic energy (red line) as properties for prediction by using LDR.

**Figure 3:**
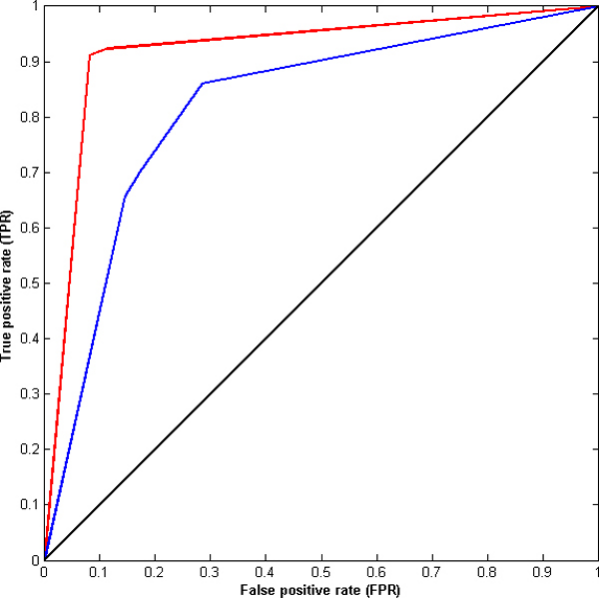
**ROC curve for the ZH-AT dataset**. ROC curves for the ZH-AT dataset using desolvation energy (blue line) and electrostatic energy (red line) as properties for prediction by using LDR.

Figures 2 and 3 show the receiver operating characteristic (ROC) curves for the MW-AT and ZH-AT datasets using electrostatic and desolvation energies as properties for prediction by LDR. These ROC curves are plotted based on the true positive rate (TPR), aka "sensitivity", vs. the false positive rate (FPR), known as "1 - specificity", at various threshold settings. For both datasets, ZH-AT and MW-AT, the prediction performances of LDR using electrostatic energy are clearly much better than using desolvation energy for prediction. In addition, the area under the curve (AUC) for each of the above ROC curves was computed. The AUC for ZH-AT using electrostatic energy is 0.90 while using desolvation energy is 0.73. Similarly, the AUC for MW-AT using electrostatic energy is 0.91 while using desolvation energy is 0.72. By comparing the AUC values, it can be concluded that electrostatic energy clearly shows much better prediction accuracy than desolvation energy.

### Analysis of distance cutoffs

In order to obtain a better insight into the classification results by using desolvation and electrostatic energies as properties, different experiments were performed by varying the distance cutoff between atom pairs of interacting chains.

Table 3 shows the prediction results for all cutoff values ranging from 5Å to 13Å for atom-type datasets, namely ZH-AT and MW-AT. For this analysis, desolvation energy values are used as the prediction properties and the NB classifier is applied for classification. The best distance cutoff for the ZH-AT dataset is 6Å, achieving 74.04% prediction accuracy, while for MW-AT the highest prediction accuracy, 77.96%, is achieved for 7Å.

**Table 3:** Prediction accuracies using desolvation energy and different distance cutoffs.

Dataset	Inter-atom distance cutoffs								
	**5Å**	**6Å**	**7Å**	**8Å**	**9Å**	**10Å**	**11Å**	**12Å**	**13Å**

ZH-AT	71.75%	74.04%	72.52%	71.75%	70.99%	69.46%	68.70%	67.93%	67.93%

MW-AT	75.99%	76.32%	77.96%	76.32%	75.99%	73.02%	73.02%	72.36%	71.38%

Prediction accuracies of NB for the ZH-AT and MW-AT datasets using desolvation energy values as the prediction properties and different distance cutoffs ranging from 5Å to 13Å.

**Figure 4:**
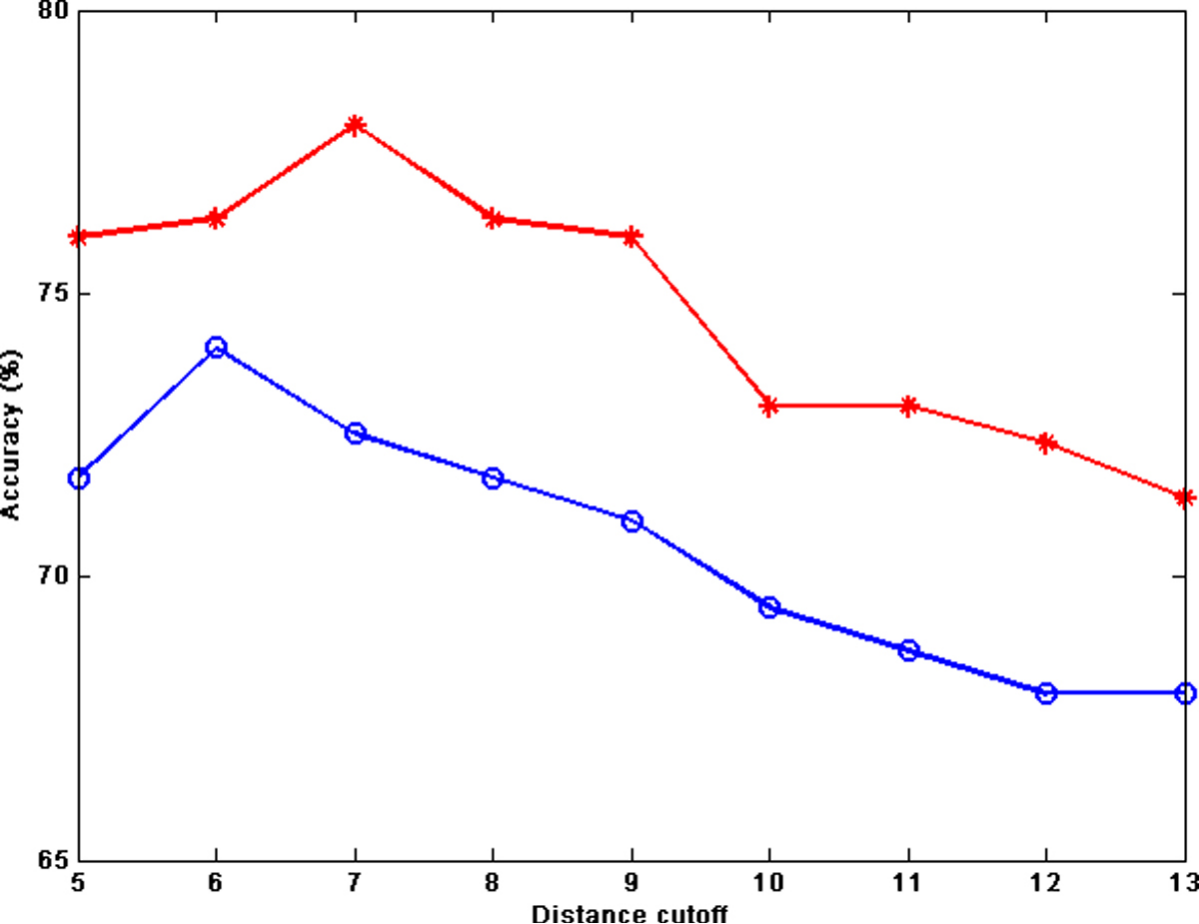
**Prediction performance using desolvation energy and different distance cutoffs**. Prediction accuracy for NB on MW-AT (red line) and ZH-AT (blue line) using desolvation energy as the prediction property and different distance cutoffs ranging from 5Å to 13Å.

In Figure 4, the performances of NB for atom type features for the MW and ZH datasets, when using desolvation energy, are plotted against the interaction distances. From the plots, it is observable that for both datasets, the best prediction accuracies are obtained for distance cutoffs between 5Å and 8Å. Moreover, for both datasets the performances decrease gradually by increasing the distance cutoffs. These results demonstrate that the best distance cutoffs for prediction by using desolvation energy is less than 8Å.

Similarly, Table 4 shows the prediction results for the ZH-AT and MW-AT datasets for distance cutoffs from 7Å to 13Å. Here, electrostatic energy is used as the prediction property and NB for classification. For the ZH-AT dataset, the best accuracy, 96.95%, is obtained for a distance cutoff of 12Å, while for the MW-AT dataset the best accuracy, 90.42%, is achieved for a distance cutoff of 11Å.

**Table 4:** Prediction accuracies for electrostatic energy and different distance cutoffs

Dataset	Inter-atom distance cutoffs						
	**7Å**	**8Å**	**9Å**	**10Å**	**11Å**	**12Å**	**13Å**

ZH-AT	94.65%	94.65%	94.65%	96.15%	96.18%	96.95%	90%

MW-AT	84.44%	84.16%	89.44%	89.44%	90.42%	89.85%	82.83%

Prediction accuracies of NB for the ZH-AT and MW-AT datasets using electrostatic energy values as the prediction properties and different distance cutoffs ranging from 7Å to 13Å.

**Figure 5:**
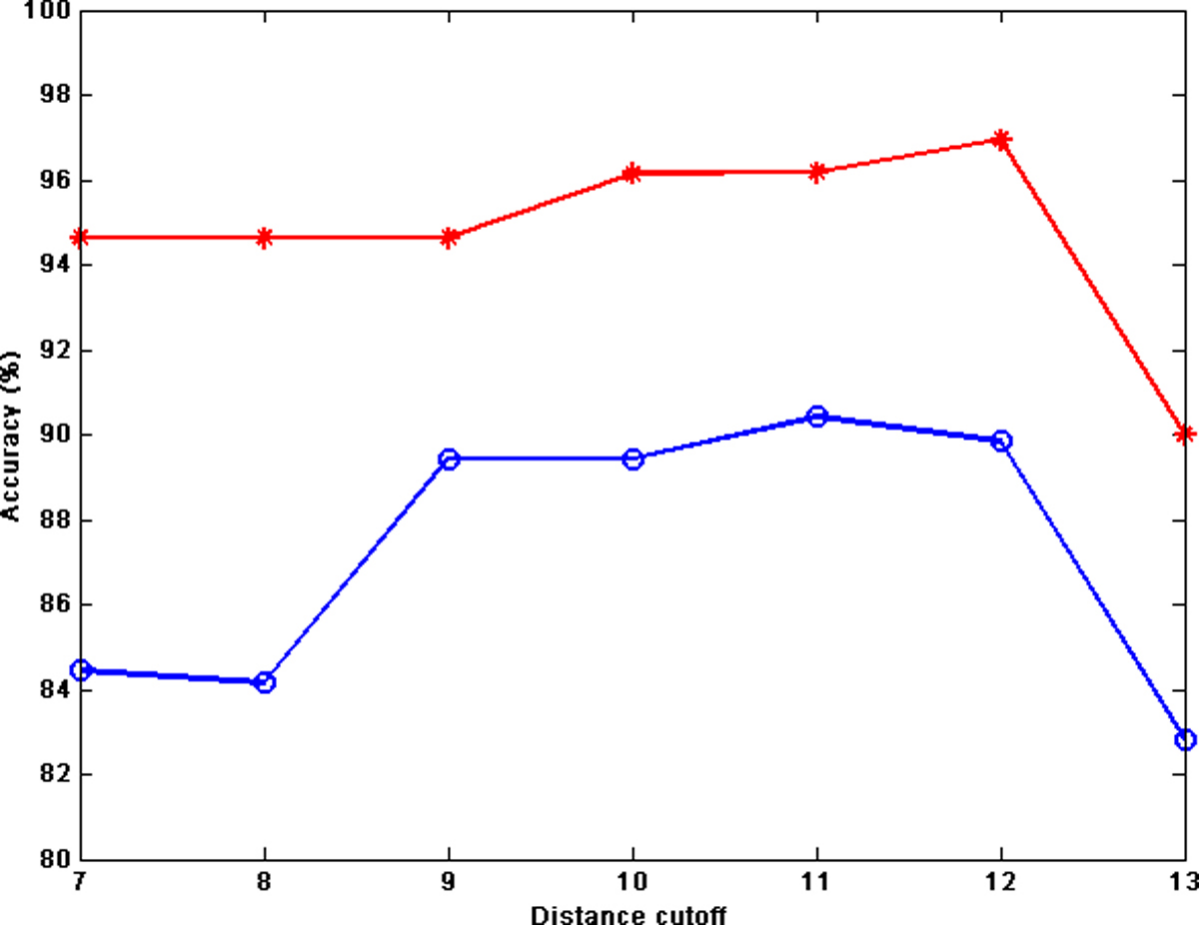
**Prediction performance using electrostatic energy and different distance cutoffs**. Prediction accuracy for NB on MW-AT (red line) and ZH-AT (blue line) using electrostatic energy as the prediction property and different distance cutoffs ranging from 7Å to 13Å.

The classification accuracies for the atom type datasets, MW-AT and ZH-AT, when using electrostatic energy, are plotted in Figure 5. The *x*-axis shows the distance cutoff between atom pairs of interacting chains (ranging from 7Å to 13Å) while the *y*-axis shows the prediction accuracy. For ZH-AT, the best accuracies are achieved for distance cutoffs in the range 10Å to 12Å, and these accuracies are all close to 96%. By increasing the distance cutoff to 13Å, the accuracy decreases rather quickly. Also, for MW-AT, the prediction accuracies in the range 9Å to 12Å are almost the same, around 90%. As in the ZH-AT, the performance decreases when the distance cutoff is increased to 13Å.

As a general remark, it can be concluded that the best distance cutoffs for prediction of obligate and non-obligate complexes using electrostatic energy range from 9Å to 12Å, while by using desolvation energy the best distance cutoffs range from 5Å to 7Å. These distance cutoffs for desolvation energy are reasonable and are in agreement with all previous studies [5,6,36]. In most studies, a distance cutoff of 6Å is typically used to determine whether or not two atoms from different chains interact with each other. Moreover, in [20,35,36], a function *g *is used to compute the distance between two atoms. These approaches consider a smooth function for inter-atom distances between 5Å and 7Å, while *g *evaluates to 0 if the distance is greater than 7Å. On the other hand, electrostatic energy is considered to be long-range [21,48], extending inter-atom interactions up to a 10°A distance or more, and hence covering a much broader and deeper area of the interface. In other words, this suggests that using electrostatic energy with a long distance cutoff, the atoms in the surface and some atoms buried under the surface may participate in the prediction that led to outstanding classification performance. This is a topic of interest for further studies.

### Analysis of feature selection

**Figure 6:**
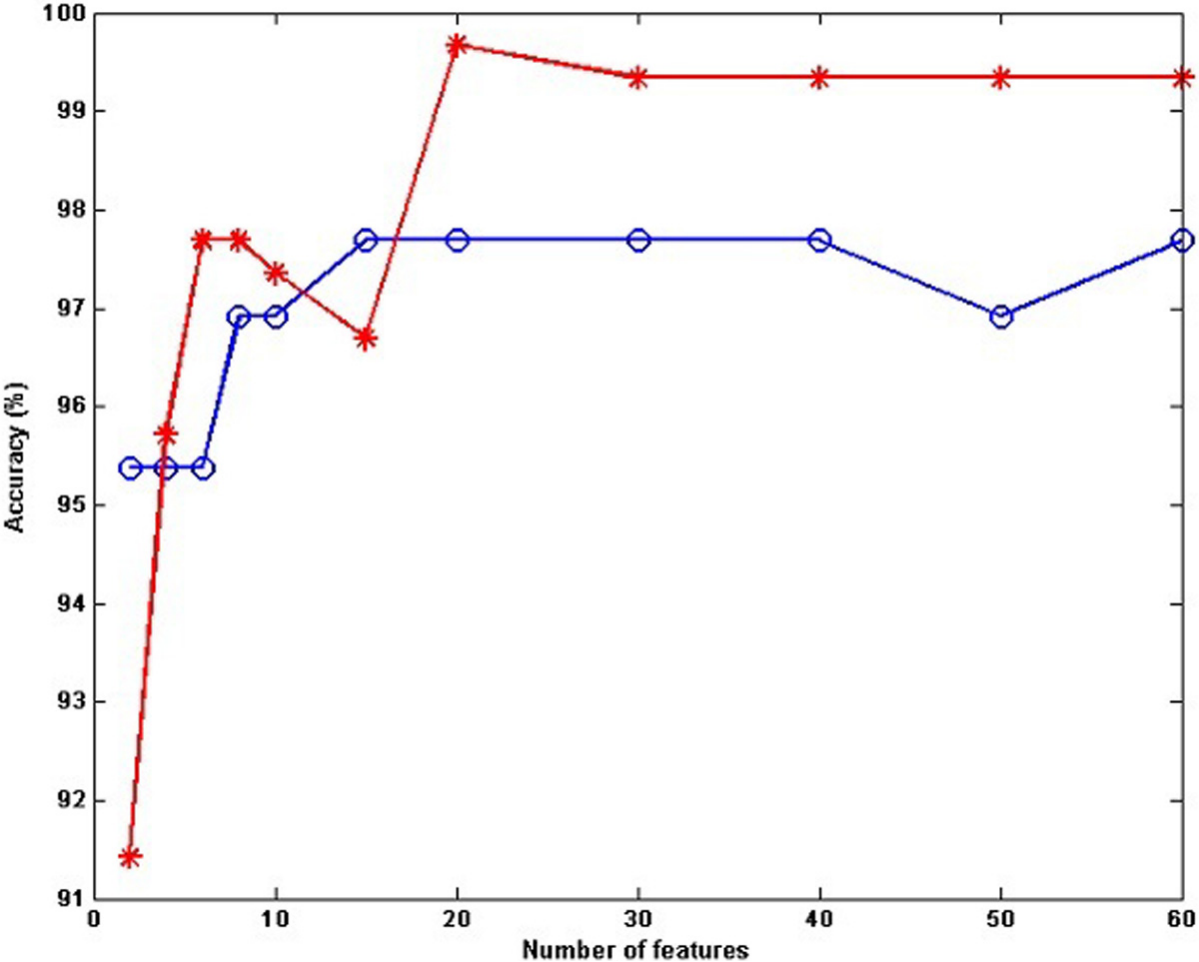
**Prediction accuracy of the ZH-AT and MW-AT datasets using GR feature selection**. Prediction accuracy for LDR on MW-AT (red line) and ZH-AT (blue line) using electrostatic energy plotted against the number of features selected by GR.

Determining the minimum number of features while keeping, or even improving, classification performance is the main challenge in all feature selection methods. To demonstrate this, the accuracies of LDR for atom type features of the MW and ZH datasets are plotted against the number of selected features in Figure 6. The order of the selected features for prediction is based on the order of features scored by GR. The best number of features for MW-AT is 20, achieving 99.67% while for ZH-AT, 15 features are found with 97.69% accuracy. From the plot, it can be concluded that (a) a few features are good descriptors for prediction of obligate and non-obligate complexes; (b) the best number of features is different from one dataset or subset of features to another; (c) prediction accuracy for the MW-AT dataset is much higher, achieving almost perfect prediction.

To compare the performance of feature selection methods and their effects on the prediction algorithms from a different perspective, the features of all datasets were scored and ranked by GR, IG, Chi2 and mRMR, separately. Then, LDR methods were applied for prediction by selecting a subset of the top-ranked features. In this experiment, the number of selected features was less than 10, starting from two features and then adding two more features at each subsequent step. The results of the LDR classifier with atom and amino acid type features with and without feature selection are depicted in Table 5. For all datasets, except MW-AA, the predictions show better performance by using feature selection methods. The most significant increase in prediction performance is for ZH-AT, for which by using only two of the top-ranked features scored by Chi2, yields 97.69% accuracy, which is much higher than using no feature selection at all (95.42%). While the most notable decrease in prediction accuracy between with and without feature selection is approximately 3%, which is observed in MW-AA by applying GR, this decrease can be acceptable considering that only four out of the 210 original features are used for prediction. This also implies savings in the required classification time and space resources.

**Table 5:** Prediction accuracies for electrostatic energy and different feature selection methods.

FS method	ZH-AA		ZH-AT		MW-AA		MW-AT	
	
	n	accuracy	n	accuracy	n	accuracy	n	accuracy
No FS	210	93.89%	171	95.42%	210	92.08%	171	96.30%

Chi2	8	97.69%	2	97.69%	10	91.09%	6	97.69%

GR	4	96.92%	8	96.92%	4	86.80%	6	97.69%

IG	8	97.69%	2	97.69%	8	88.78%	10	96.37%

mRMR	10	96.15%	10	97.69%	10	90.94%	10	96.10%

Prediction accuracies of LDR for all subsets of features (ZH-AT, ZH-AA, MW-AT and MW-AA) using electrostatic energy values as the prediction properties and different feature selection (FS) methods; *n *indicates the number of features.

In general, it can be concluded that a few pairs of atoms/amino acids are appropriate for prediction. Also, feature selection increases the performance of classification models by eliminating redundant, irrelevant and noisy features and selecting the more discriminative features. Moreover, by comparing the performance of the applied feature selection methods, Chi2 is the best method for ranking features. In contrast, mRMR is the worst ranking method because it used more features and achieved lower performance for all datasets.

### Visual analysis

To show the effect of using electrostatic energy for prediction of PPI types from a different perspective, a visual analysis is presented. In this analysis, an obligate complex, PDB ID 2min, and a non-obligate complex, PDB ID 1a2k, both from the MW dataset are considered. For these protein complexes the solvent accessible surfaces by electrostatic potential are generated with the help of Jmol embedded in APBS. In the plots, positive electrostatic potentials are shown in blue, while negative electrostatic potentials are shown in red.

**Figure 7:**
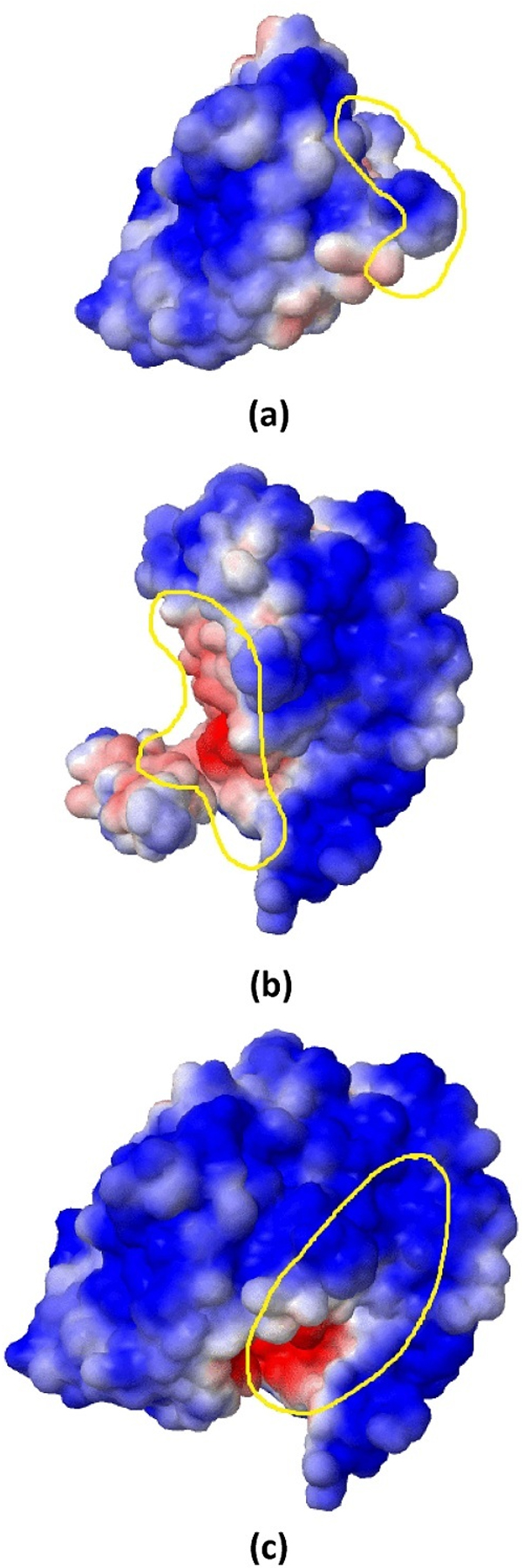
**Plot of solvent accessible surface by electrostatic potential of an obligate complex, PDB-ID **2min**, before and after the interaction takes place**. (a) Electrostatic potential of chain A of 2min, (b) Electrostatic potential of chain B of 2min, (c) Electrostatic potential of chains A and B of 2min. The plots were generated by Jmol embedded in APBS.

The electrostatic potentials of the sub-units corresponding to chains A and B of *2min *are shown in Figures 7(a) and 7(b), respectively. The whole complex (chains A and B together) is shown in Figure 7(c). By observing Figure 7(c), it is clear that the interaction between chains A and B of 2min takes place at regions of the two chains (highlighted in yellow) that have different electrostatic potentials; the highlighted region of chain A has positive charge (Figure 7(a)), while for chain B has negative charge (Figure 7(b)). It other words, the positive and negative potentials on the interface areas of chains A and B cause them to interact with each other.

**Figure 8:**
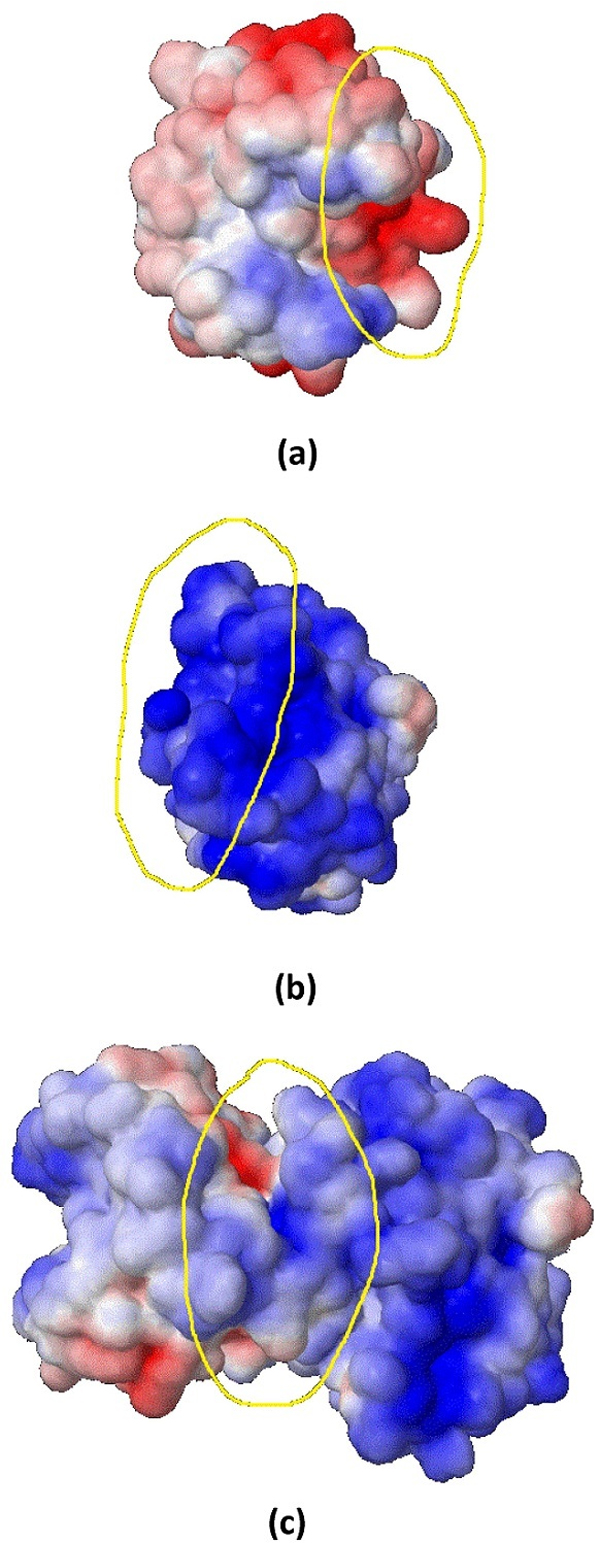
**Plot of solvent accessible surface area by electrostatic potential of a non-obligate complex, PDB-ID **1a2k**, before and after the interaction takes place**. (a) Electrostatic potential of chains AB of 1a2k, (b) Electrostatic potential of chain C of 1a2k, (c) Electrostatic potential of chains AB and C of 1a2k. The plots were generated by Jmol embedded in APBS.

Similarly, Figure 8 shows a non-obligate complex, PDB ID 1a2k AB:C, along with the electrostatic potential for three different cases: chains AB as a sub-unit (Figure 8(a)), chain C as a sub-unit (Figure 8(b)) and the whole complex including chains AB and chain C (Figure 8(c)). From the plots, it is clear that the region highlighted in yellow in Figure 8(a) shows negative electrostatic potential (shown in red), while in Figure 8(b), the highlighted yellow region shows positive electrostatic potential (shown in blue). The interaction between the two chains takes place at these regions is shown in Figure 8(c). Similarly, the positive and negative potentials on the interface areas of chains AB and chain C yield very high affinity and cause them to interact with each other. However, the interface area of complex 1a2k, which is non-obligate, is smaller than the interface area of complex 2min, which is obligate. Electrostatic energy is a very good property in the sense that it captures the size of the interface area and the complementarity of the sub-units participating in the interaction. Observing Figure 7(b), it is clear that the concavity of the sub-unit corresponding to chain B will match very well the salient part on the right of the sub-unit of chain A. These features are well captured by electrostatic energy and this is, indeed, the main aspect that we exploit to predict the stability of protein complexes, which is corroborated in the experimental results.

## Conclusions

The proposed prediction model works exceptionally well for distinguishing protein interaction types. Our prediction approach uses electrostatic energy values for pairs of atoms or amino acids present in the interfaces of obligate and non-obligate complexes. The classification is performed via various classification techniques including LDR, SVM, *k*-NN and NB.

We observe that electrostatic energy values with distance cutoffs in the range 9Å to 12Å turn out to be the best ones for prediction of interaction types on the basis of our experimental results. The reason for why electrostatic energy yields better prediction results is because electrostatic interactions are long-range. Thus, by using electrostatic energy with a large distance cutoff, not only the atoms in the surface but also some atoms which are buried under the surface may participate in the interaction, and this leads to excellent prediction results. Therefore, among various types of molecular interactions, electrostatic interactions play a special role. The proposed features then exploit the high affinity of proteins to interact with each other (in terms of negative and positive potentials). Furthermore, applying several feature selection algorithms on the MW and ZH datasets demonstrates that removing irrelevant and noisy pairs of atom type/amino acid type features and selecting the most relevant pairs improve the prediction results.

From this study, various open questions remain to be answered. One of these is to investigate domains and motifs present in the interface in order to achieve a better insight on proteins, their interactions, and function. Another problem that deserves attention is to investigate the role of buried atoms and their influence in obligate interactions. This study could consider atoms that are 10Å (or more) apart from each other, but one of these atoms may not be on the surface of the protein.

## Authors' contributions

MM carried out the computational experiments for prediction, feature selection and ROC analysis, and participated in writing the paper. GV performed all calculations of electrostatic energy values, generated electrostatic potential plots, and participated in writing the paper. LR conceived the main model for prediction using electrostatic energy, participated in writing the paper, and coordinated all tasks for the study. All authors read and approved the final manuscript.
